# Highly Sensitive *In Vivo* Imaging of *Trypanosoma brucei* Expressing “Red-Shifted” Luciferase

**DOI:** 10.1371/journal.pntd.0002571

**Published:** 2013-11-21

**Authors:** Alex P. McLatchie, Hollie Burrell-Saward, Elmarie Myburgh, Michael D. Lewis, Theresa H. Ward, Jeremy C. Mottram, Simon L. Croft, John M. Kelly, Martin C. Taylor

**Affiliations:** 1 Faculty of Infectious and Tropical Diseases, London School of Hygiene and Tropical Medicine, London, United Kingdom; 2 Wellcome Trust Centre for Molecular Parasitology, Institute of Infection, Immunity and Inflammation, University of Glasgow, Glasgow, United Kingdom; New York University School of Medicine, United States of America

## Abstract

**Background:**

Human African trypanosomiasis is caused by infection with parasites of the *Trypanosoma brucei* species complex, and threatens over 70 million people in sub-Saharan Africa. Development of new drugs is hampered by the limitations of current rodent models, particularly for stage II infections, which occur once parasites have accessed the CNS. Bioluminescence imaging of pathogens expressing firefly luciferase (emission maximum 562 nm) has been adopted in a number of *in vivo* models of disease to monitor dissemination, drug-treatment and the role of immune responses. However, lack of sensitivity in detecting deep tissue bioluminescence at wavelengths below 600 nm has restricted the wide-spread use of *in vivo* imaging to investigate infections with *T. brucei* and other trypanosomatids.

**Methodology/Principal findings:**

Here, we report a system that allows the detection of fewer than 100 bioluminescent *T. brucei* parasites in a murine model. As a reporter, we used a codon-optimised red-shifted *Photinus pyralis* luciferase (PpyRE9H) with a peak emission of 617 nm. Maximal expression was obtained following targeted integration of the gene, flanked by an upstream 5′-*variant surface glycoprotein* untranslated region (UTR) and a downstream 3′-*tubulin* UTR, into a *T. brucei* ribosomal DNA locus. Expression was stable in the absence of selective drug for at least 3 months and was not associated with detectable phenotypic changes. Parasite dissemination and drug efficacy could be monitored in real time, and brain infections were readily detectable. The level of sensitivity *in vivo* was significantly greater than achievable with a yellow firefly luciferase reporter.

**Conclusions/Significance:**

The optimised bioluminescent reporter line described here will significantly enhance the application of *in vivo* imaging to study stage II African trypanosomiasis in murine models. The greatly increased sensitivity provides a new framework for investigating host-parasite relationships, particularly in the context of CNS infections. It should be ideally suited to drug evaluation programmes.

## Author Summary

Parasites of the *Trypanosoma brucei* species complex are the causative agents of human African trypanosomiasis. There is an urgent need for new drugs to treat this debilitating and potentially fatal infection, especially in its late stage, when parasites have entered the central nervous system. Factors which hamper drug development include the limitations of the current murine models for stage II disease. *In vivo* bioluminescence imaging is a non-invasive technique that can be used to monitor infections in real time and is a powerful new approach for studying drug effectiveness. However, application of this imaging technology to trypanosome infections has been restricted because of lack of sensitivity. In this paper, we have taken a major step to resolve this problem. The enhanced sensitivity in infected mice is based on the high level expression in trypanosomes of a “red-shifted” luciferase variant that greatly improves bioluminescence detection in deep tissue. The system which we have developed should be a widely applicable tool for providing new insights into the infection biology of *T. brucei*.

## Introduction

African sleeping sickness, or human African trypanosomiasis (HAT), currently infects around 10,000 people per year and threatens the lives of a further 70 million people living in 36 countries of sub-Saharan Africa [1]. Infections of domestic livestock are also a major economic problem throughout the region. HAT is caused by protozoan parasites of the *Trypanosoma brucei* species complex which are transmitted to mammalian hosts by the tsetse fly during a blood meal. There are two sub-species of human infective parasite. *Trypanosoma brucei gambiense*, which is responsible for 90% of clinical cases, causes a chronic form of the disease. *T. brucei rhodesiense*, which is primarily zoonotic, causes an acute form of HAT in eastern Africa. The sub-species *Trypanosoma brucei brucei* is non-human infectious, but is a pathogen of domestic animals.

HAT has two distinct stages. The first is characterised by recurrent fever and malaise as the parasite replicates in the blood and lymph. The second begins once parasites penetrate the blood brain barrier and access the central nervous system (CNS). This can occur weeks (*T.b. rhodesiense*) or years (*T.b. gambiense*) after the initial infection. Without chemotherapeutic intervention, this leads rapidly to neurological impairment, coma, and death. The drugs currently used to treat HAT can be difficult to administer, are associated with a range of side effects, and treatment failures are frequently reported [2], [3]. The front line drugs, pentamidine and suramin, were developed over 50 years ago and are only useful against the haemo-lymphatic stage of disease. Pentamidine is the drug of choice against *T.b. gambiense*, while suramin is only employed to treat *T.b. rhodesiense* infections. Melarsoprol and eflornithine, are both used to treat the second stage of infection following CNS invasion, but both have significant disadvantages. Melarsoprol is directly associated with encephalopathic syndrome, which arises in 5–10% of cases and has a mortality rate of 50–70% [4], [5]. Eflornithine causes less severe side effects, but it is expensive and difficult to administer, requiring four infusions of 400 mg kg^−1^ each day over a 7 to 14 day period, and is not used against *T.b. rhodesiense*. More recently, a nifurtimox and eflornithine combination therapy (NECT) has been introduced that is more effective against late stage *T.b. gambiense* infections than eflornithine monotherapy. With increasing accounts of clinical relapse and the growing likelihood of drug resistance, there is an urgent need to develop novel drugs that are safe, effective during stage II CNS disease, and easy to administer in the field.

A current murine model of chronic stage II trypanosomiasis utilises the pleomorphic *T.b. brucei* GVR35 strain [6]. However, *in vivo* evaluation of compounds for trypanocidal activity using this system is both labour and animal intensive and requires 180 days post-treatment to completely assess efficacy. Non-invasive whole body bioluminescence imaging can be utilized as an alternative approach in a variety of *in vivo* disease models to accelerate drug discovery and development [7]–[9]. A particular advantage of bioluminescence imaging is that the detected signal can be directly correlated with pathogen load. This provides a simple, non-invasive way of quantifying the effect of therapeutic intervention in real time. The use of a stage II trypanosomiasis murine model that incorporates easily detectable bioluminescent parasites, would greatly streamline the drug testing process, particularly when assessing activity against late stage brain infections.

One limitation of bioluminescence imaging results from the loss of optical resolution of parasites situated in deep tissue. This is caused by the absorption and scatter of light which decreases the detectable signal by up to ten fold per centimetre of tissue [10]. Haemoglobin is primarily responsible for this phenomenon in mammals. However, because haemoglobin absorbs light mostly in the visible blue-green spectrum, this loss of sensitivity can be reduced by using red-shifted bioluminescent reporters that emit light above 600 nm [11], [12]. Previous reports of *in vivo* imaging of *T. brucei* infections were based on expression of *Renilla* luciferase, which emits light in the 480 nm range [13], [14], and firefly luciferase with emission at 562 nm [15].

Here, we report the development and optimisation of *T.b. brucei* Lister 427 and *T.b. brucei* GVR35 reporter cell lines that constitutively express a red-shifted mutant firefly luciferase with an emission maximum of 617 nm. These parasites are detectable in deep tissue at numbers considerably lower than previously reported and will have numerous *in vivo* imaging applications.

## Materials and Methods

### Ethics statement

BALB/c and C57BL/6N studies were carried out under UK Home Office regulations (Project licence PPL 70/6997). The CD-1 mouse work was performed with the approval of the University of Glasgow Ethics Committee (Project licence PPL 60/4442).

### Parasite culture and transfection

*T.b. brucei* Lister 427, MITat 1.2 (clone 221a) bloodstream forms were cultured and maintained *in vitro* with HMI-9 medium [16], supplemented with 10% foetal bovine serum (FBS) at 37°C in 5% CO_2_. *T.b. brucei* GVR35 bloodstream forms were cultured and maintained in HMI-9 medium supplemented with 20% FBS, 20% serum plus (Sigma) and 0.1% methyl cellulose. Both strains were electroporated with the Amaxa Nucleofector II, using program X-001 and human T-cell nucleofector buffer, and 10 µg (Lister 427), or 30 µg (GVR35) of linearised construct DNA. Transgenic bloodstream forms were selected and maintained with 1 µg ml^−1^ puromycin.

### Construct design and development

**Figure 1:**
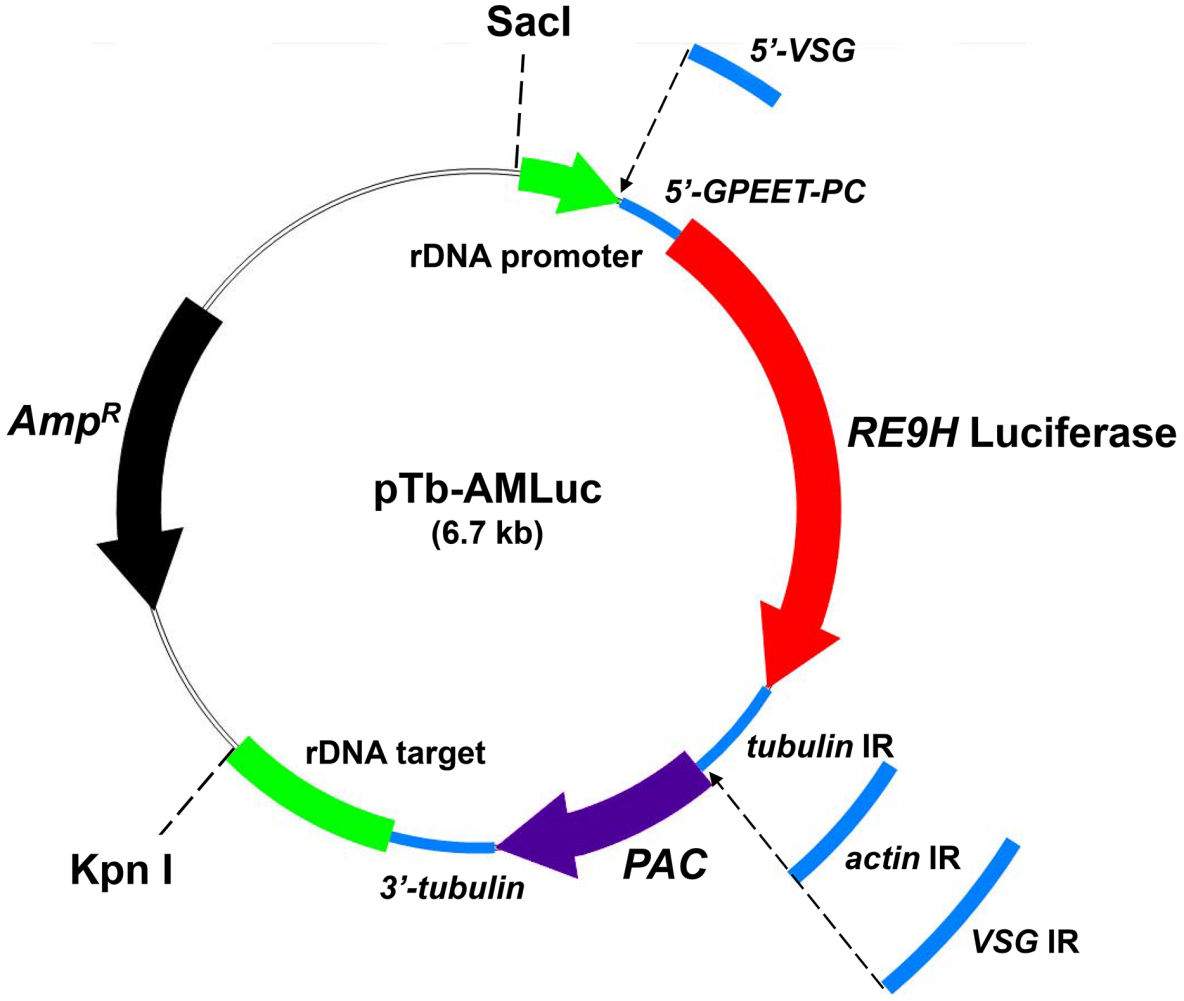
Schematic of pTb-AMluc constructs containing luciferase reporter genes. We used a modular format for the construction of the pTb-AMluc series of vectors that were designed to target the “red-shifted” luciferase genes *PpyRE9H* and *PpyRE-TS* (red) to the rDNA locus of *T.b. brucei*. The ribosomal locus targeting fragments (green), and *VSG*, *GPEET2 procyclin* and *tubulin* flanking sequences (blue) were derived from parasite DNA by PCR as outlined in Materials and Methods and Supplementary Table 1. IR refers to the intergenic region that the contains processing signal(s) at the 5′-end of the puromycin N-acetyl transferase (*PAC*) (purple) and the 3′-end of the *REH9* luciferase genes. Constructs were linearised by SacI/KpnI digestion prior to transfection.

To generate the parental integration construct pTb-AMluc (Figure 1), a 250 bp fragment derived from the *T.b. brucei* ribosomal DNA (rDNA) promoter was amplified from genomic DNA using a forward primer that introduced a 5′-SacI site and a reverse primer that introduced 3′-MluI and NotI sites (all primers used are described in Table S1). In addition, a 563 bp fragment from the rDNA non-transcribed spacer region was amplified using a forward primer that introduced a 5′-ApaI site and a reverse primer that introduced a 3′-KpnI site. The amplicons were digested with SacI/NotI and ApaI/KpnI, respectively, and ligated sequentially either side of a puromycin resistance cassette in a pBluescript backbone (Figure 1). To test 5′-untranslated regions (UTR), fragments derived from *T.b. brucei VSG221* (198 bp) and *T.b. brucei GPEET2 procyclin* (89 bp) genes were amplified using forward primers that introduced 5′-NotI sites and reverse primers that introduced 3′-XhoI sites. Each amplicon was NotI/XhoI digested and ligated into separate pTb-AMluc vectors. To test 3′-UTR regions, fragments from the corresponding regions of *T.b. brucei VSG221* (198 bp) and a*ctin A* (289 bp), were amplified using forward primers that introduced BamHI sites and reverse primers that introduced HindIII sites. Each 3′-UTR amplicon was BamHI/HindIII digested and ligated into the corresponding vectors. The red-shifted luciferase genes *PpyRE9H* and *PpyRE-TS* [12] were amplified with a 5′-forward primer that introduced an XhoI site and a reverse primer that introduced a 3′-BamHI site. Each reporter gene fragment was XhoI/BamHI digested and ligated into separate backbones, to create the final constructs (Figure 1). Prior to transfection, vectors were linearised by SacI/KpnI digestion.

### *In vitro* luciferase assays, parasite growth curves and analysis of construct stability

Luciferase assays were performed with a kit according to the manufacturer's protocol (Promega). Prior to each assay, cells were grown to mid-logarithmic phase and 5×10^6^ cells were pelleted by centrifugation. The cells were lysed by resuspension in 50 µl of CCLR buffer (Promega). 10 µl of this was added to 100 µl of luciferase substrate. End point or spectral luminescent readings were taken using a SpectraMax M3 Microplate Reader (Molecular Devices GmbH). Growth was monitored by counting each cloned cell line on consecutive days over a 12 day period, followed by daily dilution back to 1×10^5^ parasites ml^−1^. Construct stability was tested by culturing each clone in the presence or absence of 1 µg ml^−1^ puromycin and assaying luciferase activity weekly over a period of three months.

### Mice infection, imaging and welfare

Female BALB/c, CD1 and C57BL/6N mice were purchased from Charles River (MA., USA) and housed in individually ventilated cages. For infection studies, mice (18–20 g) were inoculated intraperitoneally (i.p.) with parasites in 200 µl HMI9 medium. For imaging, mice were inoculated i.p. with 200 µl D-luciferin (15 mg ml^−1^ in Mg/Ca-free Dulbecco's modified PBS) (Perkin Elmer), and 10 minutes later anaesthetised with 2.5% isofluorane. Light emission was recorded using Lumina or Spectrum *in vivo* imaging systems (IVIS) (Perkin Elmer). Exposure times varied between 1 second and 5 minutes, depending on signal intensity. Peripheral parasitemia was scored by counting Giemsa-stained smears of tail blood. To clear peripheral parasitemia, mice were treated with a single 40 mg kg^−1^ oral or i.p. dose of berenil (1,3-Bis(4′-amidinophenyl)triazene) and imaged between 4 and 7 days later. Mouse brains were excised after perfusion for *ex-vivo* imaging. Briefly, mice were exsanguinated under terminal anaesthesia, then underwent vascular perfusion via the hepatic portal vein with 10 ml PBS. Successful perfusion was assessed by blanching of the liver. The whole brains were then excised and imaged in the Lumina IVIS. To enhance the signal and avoid desiccation, approximately 100 µl D-luciferin (15 mg ml^−1^ in Mg/Ca-free Dulbecco's modified PBS) was pipetted onto the surface of each brain 5–10 minutes prior to imaging [15], [17]. In accordance with local animal welfare regulations, infected mice were euthanised when they displayed immobility, hind-leg paralysis, or 20% weight loss.

## Results

### Construct optimisation

**Figure 2:**
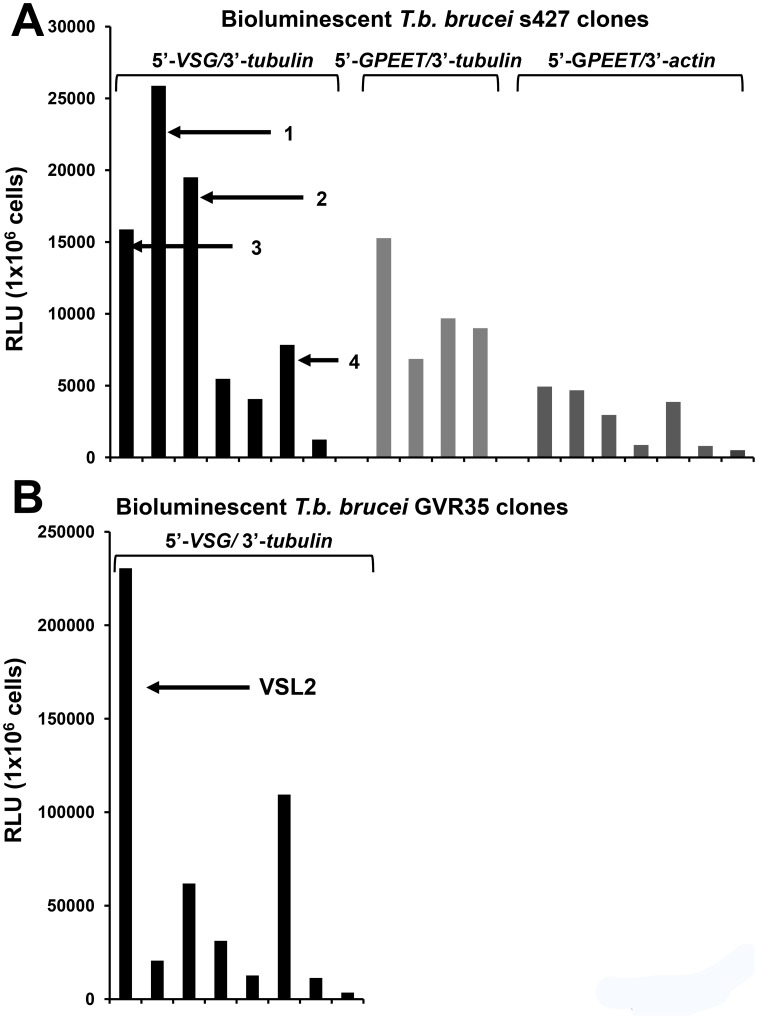
Isolation of *T.b. brucei* clones expressing high level bioluminescence. (**A**) The bioluminescent signal (RLU) detected from *T.b. brucei* s427 clones transfected with pTb-AMluc constructs in which the *PpyRE9H* gene was flanked by 5′-*VSG* and 3′-*tubulin*, *5′-GPEET2 procyclin* and 3′-*tubulin*, or 5′-*GPEET2 procyclin* and 3′-*actin* UTRs (Figure 1). Luciferase assays were carried out on 10^6^ cells (Materials and Methods), with readings taken using a SpectraMax M3 Microplate Reader. The data show that the 5′-*VSG*/3′-*tubulin* UTR combination in construct pTb-AMluc-v gives the highest luciferase signal *in vitro*. Clones 1–4 (indicated) were used in assessment of growth (Figure S1). (**B**) The bioluminescent signal detected from *T.b. brucei* GVR35 clones transfected with construct pTb-AMluc-v. Clone VSL2 (indicated) was chosen for *in vivo* studies.

In trypanosomes, protein coding genes can be transcribed by RNA polymerase I (Pol-I), leading to high levels of expression [18]. We therefore designed integration vectors where the bioluminescent reporter genes would be targeted to the rDNA loci, under the control of a Pol-I dependent rDNA promoter (Figure 1). Constructs containing the red-shifted luciferase genes *PpyRE-TS* (thermostable) and its derivative *PpyRE9H* (humanised codon usage) [12], flanked by the 5′-*GPEET2 procyclin* splice site and 3′-*tubulin* UTR sequences, were first used to transfect bloodstream form *T.b. brucei* Lister 427. This is a monomorphic strain in which transformants can be generated in only 5–7 days. Parasite clones expressing PpyRE9H were found to display much higher luciferase activities than those expressing PpyRE-TS (data not shown). A series of constructs was then generated to identify the combination of 5′- and 3′-UTR regions which facilitated the optimal level of PpyRE9H expression (Figure 1). Analysis of transformed clones revealed a range of luciferase activities in each case (Figure 2A). Interestingly, replacement of the 3′-*tubulin* intergenic sequence with the corresponding sequence from the *actin A* gene, resulted in a general reduction of expression levels in transfected trypanosomes. The highest expression levels were found in parasites transfected with a construct (pTb-AMluc-v) containing the 5′-*VSG* and 3′-*tubulin* UTR pairing. We next transfected the pleomorphic *T.b. brucei* GVR35 strain with the pTb-AMluc-v construct. Again, we observed a wide range of expression levels (Figure 2B). One clone (VSL2) expressed luciferase activity that was almost 10-fold greater than the highest expressing Lister 427 clone.

To establish if integration and expression of the reporter gene affected parasite growth, GVR35 and Lister 427 clones were followed in culture. There were no significant differences in the growth rate of transgenic and wild type cell lines (Figure S1). To ensure that reporter gene expression was stable over time, cell lines were sub-cultured in the absence of selective drug pressure for up to 3 months. This time span equates to more than 100 generations in the pleomorphic GVR35 cell line and 300 generations in the monomorphic 427 cell line. No significant changes in bioluminescence were detected (Figure S2A and B).

### Limit of detection *in vitro* and *in vivo*

**Figure 3:**
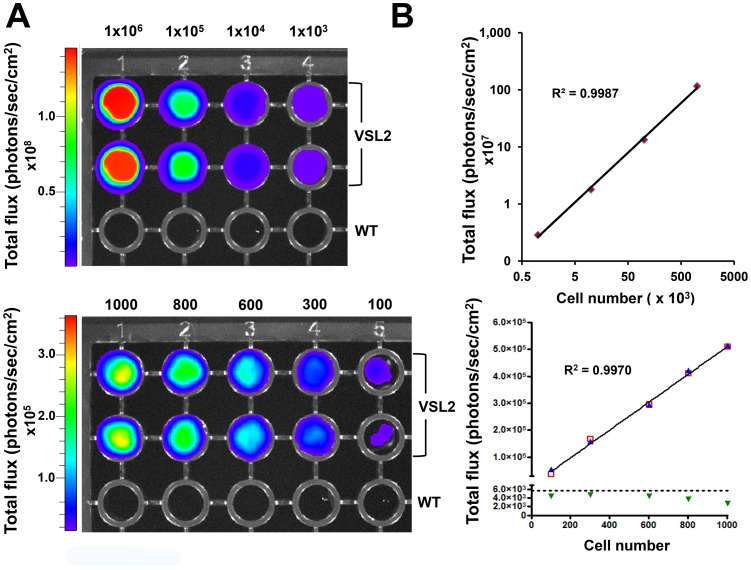
Limit of detection *in vitro*. (**A**) Images of two 96-well microtitre plates containing dilutions of *T.b. brucei* GVR35 clone VSL2 and a non-transformed control (WT). Each plate was imaged using an IVIS Lumina (Perkin Elmer) with 1 minute exposure and medium binning. Both cell lines were serially diluted from 1×10^6^ to 1×10^3^ (upper plate) and from 1×10^3^ to 1×10^2^ parasites ml^−1^ (cell numbers shown above each plate). 100 VSL2 parasites could be clearly visualised. Note that the imaging software automatically adjusts the heat-map scale to account for the intensity of the well containing the highest number of parasites. (**B**) *In vitro* linear regression plots generated from both plates. Each point corresponds to bioluminescence represented by the total flux recorded from a single well. In both cases, linear regression analysis shows a very strong positive correlation between bioluminescence and parasite number (R^2^>0.99). The graphs show readings from duplicate wells. In the upper graph, duplicate values were extremely close and are not individually distinguishable. In the lower graph, duplicates are shown as red squares and blue triangles, and the dotted line indicates the background in blank wells (green triangles), plus two standard deviations.

We assessed if there was a direct correlation between bioluminescence and cell number by conducting luciferase assays on serial dilutions of the VSL2 parasite clone (Figure 3). Linear regression analysis showed a strong positive correlation between the level of bioluminescence and the number of live cells (R^2^ = >0.99). We also sought to determine the limit of detection achievable with the Lumina IVIS. Serial dilutions of the VSL2 clone in a 96-well microtitre plate format were imaged after the addition of luciferin (Materials and Methods). 100 parasites could be readily visualised (Figure 3A).

**Figure 4:**
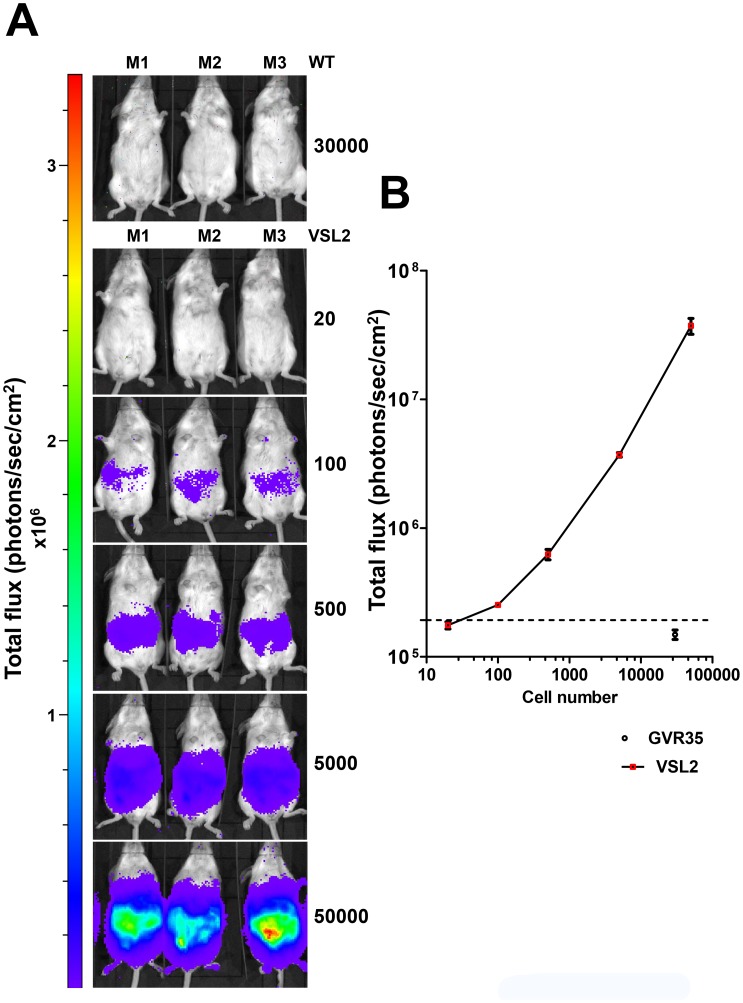
Limit of detection *in vivo*. (**A**) Six sets of three BALB/c mice were inoculated i.p. with either 20, 100, 500, 5000, or 50000 bloodstream form *T.b. brucei* GVR35 clone VSL2. An additional set of three mice was inoculated with 30000 non-transformed parasites (WT). All of the IVIS Lumina images were acquired using large binning, 5 minute exposures, 15 minutes after infection and 10 minutes after administration of luciferin (150 mg kg^−1^). It was possible to visualise as few as 100 parasites in the intra-peritoneal space. (**B**) A dose response curve generated from the *in vivo* limit of detection data. Mean abdominal bioluminescence was recorded from each group. Linear regression analysis shows a very strong positive correlation between bioluminescence and parasite inoculum (R^2^>0.99). Dotted line indicates mean bioluminescence plus two standard deviations, from mice infected with wild type parasites.

To determine the limit of detection *in vivo*, BALB/c mice were inoculated i.p. with a range of parasite loads and imaged following injection with luciferin (Figure 4A). Using a maximum exposure setting, it was possible to visualise as few as 100 parasites in the intra-peritoneal space. This level of sensitivity was between two and four orders of magnitude greater than has been reported elsewhere with trypanosomatid parasites [7], [13], [14], [19]–[21]. There was also a strong positive correlation between bioluminescence and parasite inoculum when the total flux was recorded from individual mice (Figure 4B). This suggests the feasibility of assessing in real time the extent of parasite burden over several orders of magnitude directly from bioluminescence during the course of an infection, even when parasites are undetectable by microscopic analysis of peripheral blood.

### Monitoring the course of infection and the effect of drug treatment

**Figure 5:**
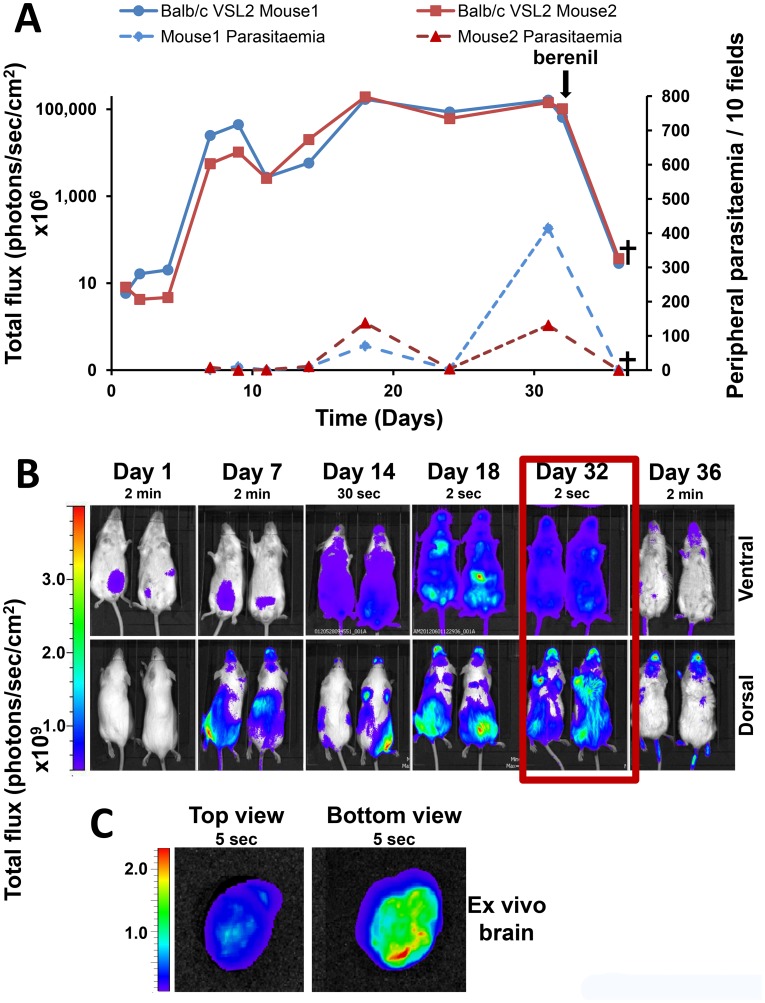
Monitoring the course of infection. (**A**) Bioluminescence (total flux) recorded from two BALB/c mice inoculated i.p. with 3×10^4^ *T.b. brucei* GVR 35 (clone VSL2) vs peripheral blood parasitemia recorded over the course of 36 days. Following infection, the bioluminescence signal fluctuated, peaking at 2×10^11^ photons/sec/cm^2^ on day 18, and remaining above 5×10^10^ photons/sec/cm^2^ until drug intervention. After treatment with berenil on day 32 (Materials and Methods), total flux and peripheral parasitemia fell rapidly. (**B**) Ventral and dorsal *in vivo* imaging of both mice (Materials and Methods) revealed the rapid growth and dissemination of parasites during the course of infection. Dorsal imaging suggests that as early as day 7, parasites can be detected in the head of both mice. After berenil treatment, the bioluminescent signal was rapidly cleared from the periphery and by day 36 was only weakly detectable. However, there was a strong focal signal localised to the head, particularly apparent when viewed from the dorsal perspective. These mice were sacrificed in accordance with animal welfare regulations. (**C**) Brains were removed from other mice 33 days post-infection and imaged after perfusion (Materials and Methods). All brains from infected animals showed a clear bioluminescent signal.

The course of infection in BALB/c mice was assessed following the inoculation i.p. with 3×10^4^ VSL2 *T.b. brucei* GVR35 parasites (Materials and Methods). This experimental model of chronic stage II trypanosomiasis is characterised by CNS infection and death after approximately 35 days, in the absence of drug treatment. The mice were imaged one day after infection and then at subsequent time points (Figure 5). At each time point, blood smears were also taken from the tail to quantify peripheral parasitemia. Bioluminescence imaging allowed the development of infection to be visualised throughout the entire experiment, whereas parasites only became detectable in peripheral blood 14–18 days post-infection (Figure 5A). The subsequent transient decrease in bloodstream parasitemia observed after this time point presumably results from antibody-mediated killing and antigenic variation within the parasite population. During this apparent clearance of infection, as judged by microscopic examination of blood, parasitemia was readily detectable by *in vivo* imaging (day 24, Figure 5A). Brain infections could be tentatively identified within 7 days of inoculation, particularly when mice were imaged from the dorsal perspective. After 32 days, the mice were treated with berenil, a drug which can resolve bloodstream, but not CNS infections [22]. As expected, there was a dramatic effect, with complete clearance of parasitemia, as judged by microscopic examination of blood smears (Figure 5A, B). With bioluminescence imaging however, it was clear that parasites had not been eradicated, with specific foci of infection in the cranial area. Examination of excised brains from other infected mice (day 33) revealed clear evidence of wide-spread infection, with distinct, highly intense bioluminescence (Figure 5C). Interestingly, bioluminescence was observable 4 days post-treatment in the region of the mouth and nose (Fig. 5B). This signal could be indicative of parasite survival in the nasal associated lymphoid tissue or result from trypanosomes which had spread from the CNS via the lymphatics into the plate region. In the absence of drug treatment, the mean survival time (Materials and Methods) for BALB/c mice infected with the bioluminescent VSL2 clone was 34.4±1.2 days (n = 14 mice).

**Figure 6:**
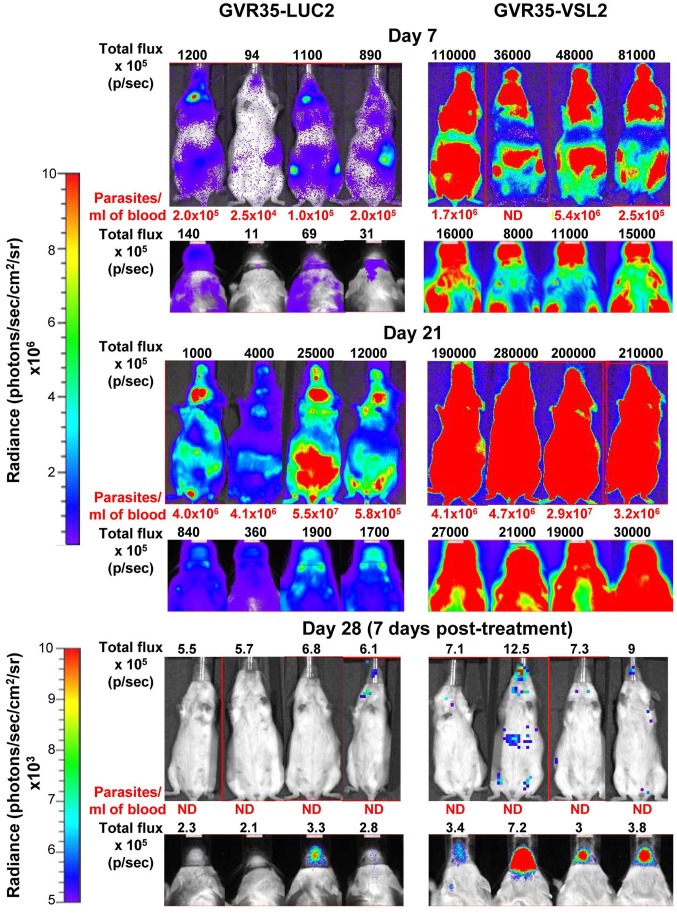
Comparison of firefly luciferase and the “red-shifted” variant as reporters *in vivo*. CD-1 mice were inoculated with 3×10^4^ bloodstream form trypanosomes expressing yellow firefly luciferase (GVR-LUC2) and the red-shifted variant (GVR35-VSL2). Ventral body (upper) and dorsal head (lower) images of mice were taken after 7 and 21 days using the IVIS Spectrum. Bioluminescence (total flux) and peripheral bloodstream parasitemia values are shown. The mice were then treated i.p. with berenil (Materials and Methods) and imaged 7 days later.

To assess the increased sensitivity achievable with the “red-shifted” luciferase, as compared to the wild type reporter gene, we carried out parallel infections of CD-1 mice. One set was infected with VSL2 *T.b. brucei* GVR35 trypanosomes and the second with parasites expressing a yellow firefly luciferase (LUC2) [15] integrated at a rDNA locus (Figure 6). Images taken 7 days and 21 days post-infection clearly show the enhanced bioluminescent signal achievable with the “red-shifted” reporter. Similarly, when mice were then treated with berenil, residual brain infections were much more readily detectable in mice infected with the VSL-2 parasites.

**Figure 7:**
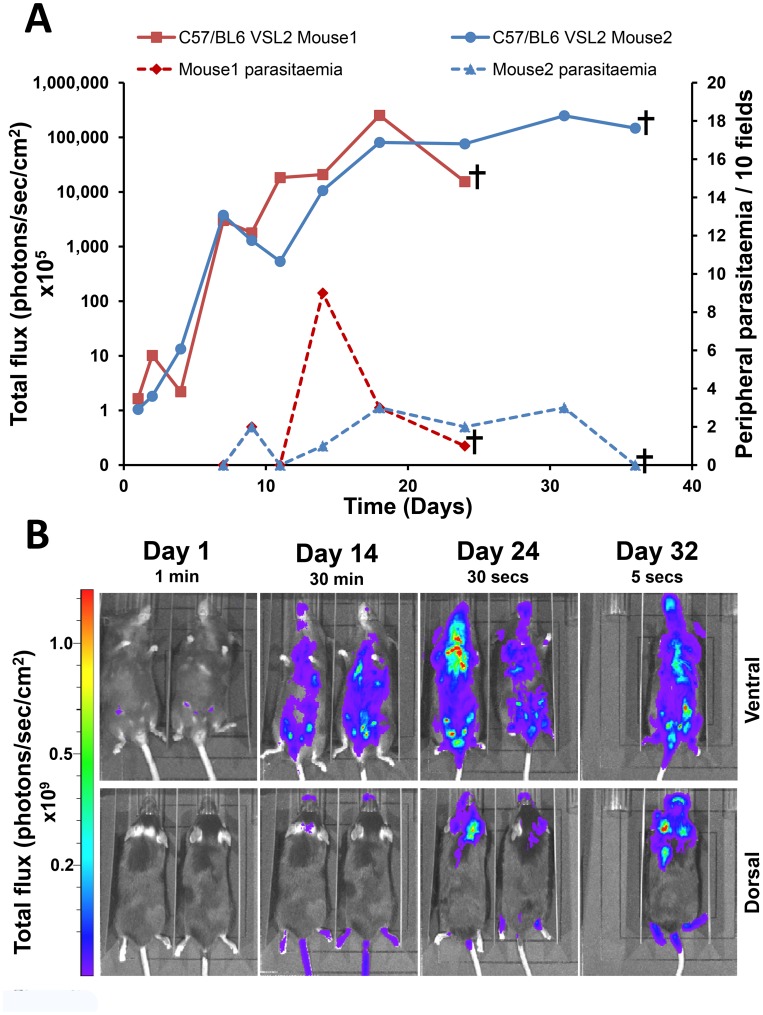
Monitoring the course of infection in C57BL/6N mice. (**A**) Bioluminescence (total flux) and peripheral blood parasitemia in mice inoculated i.p. with 3×10^4^ *T.b. brucei* GVR35 (clone VSL2). Following infection, the bioluminescence signal fluctuated, but remained above 10^10^ photons/sec/cm^2^ until termination of the experiment. Mouse 1, which developed a heavier infection, died at day 25. (**B**) Ventral and dorsal *in vivo* imaging (Materials and Methods) revealed the rapid growth and dissemination of parasites during the course of infection. Parasites can be detected in the heads of both mice by day 24.

We also examined the sensitivity of imaging when applied to C57BL/6N mice, the most commonly used strain for the generation of transgenic lines. In this background we observed that the level of bloodstream parasitemia was lower than in the BALB/c strain. In addition, there appeared to be some quenching of the signal, presumably due to the black fur of this mouse strain. Nevertheless, the course of infection could be easily monitored with the bioluminescence system and it appeared to follow a similar pattern to that in BALB/c mice (compare flux traces in Figure 5A and Figure 7A). Distinct signals appeared in the head region between day 14 and day 24 (Figure 7) corresponding to the CNS infection seen in the BALB/c mice.

## Discussion

The major goal of the work described here was to improve the tools available for *in vivo* bioluminescence imaging of trypanosome infections, as an aid to drug development programmes and studies on disease pathology. We addressed two major parameters that in combination have led to a greatly increased sensitivity of detection. First, we optimised stable high level expression of the reporter gene in transgenic parasites, and second, we used a variant luciferase protein that had been modified to enhance deep tissue detection *in vivo* [12].

The expression vector used in this study was designed to target the multi-copy rDNA loci with the bioluminescent reporter gene under the control of a ribosomal promoter (Figure 1). Previous work had shown that this strong promoter can facilitate high level expression of protein coding genes in trypanosomes [18]. In addition, we hypothesised that *in situ* transcription termination signals at the targeted locus would prevent the aberrant expression of downstream genes. We chose to avoid integration at a polycistronic Pol-II locus, such as the *tubulin* gene array, in case perturbation of local gene expression had phenotypic consequences. There are eighteen rRNA arrays in the diploid *T. brucei* genome, spread over several chromosomes [23]. When transfectants were examined for luciferase activity, we found significant differences between clones derived from the same electroporation (Figure 2). This may reflect differing levels of epigenetic control, either repression or de-repression, at the targeted rDNA loci. These observations emphasise the need to examine a number of clones for luciferase activity each time a new parasite isolate is transfected with this class of construct. As post-transcriptional control of gene expression in trypanosomatids is of particular importance [24], we also tested various sets of 5′- and 3′-UTR sequences in the context of rDNA integration, to determine the best combination for expression. As shown (Figure 2), the highest levels of expression were associated with construct pTb-AMluc-v, which contain sequences from 5′-*VSG* and 3′-*tubulin* untranslated regions. Motifs in these UTR regions of trypanosomatid genes can have a major influence on transcript processing and stability, or translational efficiency.

Most *in vivo* bioluminescence imaging studies previously published have used luciferase reporter proteins which emit light in the 480–560 nm range [7]–[9], [13]–[15]. However, this wavelength coincides with the region of the spectrum where light absorbance by haemoglobin is maximal, with a concomitant quenching of the signal when parasites are localised in deep tissue. Here, we used a thermostable luciferase variant from the North American firefly *Photinus pyralis*, which had been mutated to generate light with a peak emission wavelength of 617 nm [12]. In addition, the gene (*PpyRE9H*) had been altered to conform to human codon bias, which is similar to that of *T.b. brucei* [25], and modified further to remove non-coding repeats, local hairpins and cryptic splice sites [12]. When mice were infected with transformed bloodstream forms expressing *PpyRE9H* in the context of the integrated pTb-AMluc-v vector, it was possible to detect as few as 100 trypanosomes when concentrated in the peritoneum, with a direct correlation between parasite load and the total bioluminescent flux (Figure 4). Parasites in brain might be less readily detected due to light absorption and scattering by the skull. The pattern of infection and pathology mirrored that obtained with non-transfected *T.b. brucei* GVR35, an experimental model for stage II trypanosomiasis (Figure 5). Consistent with this, when transformed bloodstream parasites were monitored *in vitro*, there was no evidence of an effect on growth and reporter gene expression was stable for at least 3 months in the absence of selective pressure (Figure S1, S2).

The level of imaging sensitivity reported here significantly surpasses that which has been reported previously with trypanosomatid parasites [7], [13], [14], [19]–[21]. It permits infections to be followed in real time, in a non-invasive manner, even when peripheral bloodstream parasitaemia is sub-patent. The ability to image such low numbers of trypanosomes will find widespread use for monitoring chronic CNS infections where the level of parasite burden may be low. In addition, it will allow the visualisation of tissue-specific clearance of infection after drug treatment, without the need for sacrificing mice at each stage of the process (Figure 5B, as example). An additional benefit of this bioluminescent parasite line is that it is easily detectable in C57BL/6 mice (Figure 7), the background used in most transgenic experiments. With this non-invasive imaging technology, it will therefore be feasible to assess the contributions of different genes to host-parasite interactions, parasite dissemination and CNS infection, using genetically modified bioluminescent trypanosomes in combination with transgenic mouse strains. The genes encoding the “red-shifted” luciferase variants used in this study [12] should also be transferable to *T. cruzi* and *Leishmania* to enhance the applicability of imaging technology to experimental infections with these related pathogens.

## Supporting Information

Figure S1 Cumulative growth of cultured bloodstream form *T. brucei* expressing the “humanized” thermostable red-shifted luciferase (Ppy RE9H). Upper graph; *T. brucei* s427 and four independent bioluminescent clones (see Figure 2). Lower graph; *T. brucei* GVR35 and the highly expressing bioluminescent line VSL2 used in most of the imaging experiments. Parasite growth was monitored as outlined in the Materials and Methods.
(PDF)

Figure S2 Stability of luciferase expression in the absence of selective pressure. (**A**) The Ppy RE9H luciferase signal of a *T. brucei* s427 clone parasite maintained in the presence or absence of 1 µg ml^−1^ of puromycin. (**B**) The *Ppy RE9H* luciferase activity expressed by a *T. brucei* GVR35 clone. No significant decrease in bioluminescence was observed during this time, showing stable inheritance of luciferase expression. Each experiment was performed in triplicate and values shown are the means ± SD.
(PDF)

Table S1 Primer sequences.
(PDF)
