# Supplementary material for: Highly Sensitive In Vivo Imaging of Trypanosoma brucei Expressing “Red-Shifted” Luciferase
Source: PLoS Negl Trop Dis. 2013 Nov 21;7(11):e2571. doi: 10.1371/journal.pntd.0002571 (PMC3836995; doi:10.1371/journal.pntd.0002571)
Supplement: Figure S1 — Cumulative growth of cultured bloodstream form T. brucei expressing the “humanized” thermostable red-shifted luciferase (Ppy RE9H). Upper graph; T. brucei s427 and four independent bioluminescent clones (see Figure 2). Lower graph; T. brucei GVR35 and the highly expressing bioluminescent line VSL2 used in most of the imaging experiments. Parasite growth was monitored as outlined in the Materials and Methods. (PDF) [file pntd.0002571.s001.pdf]

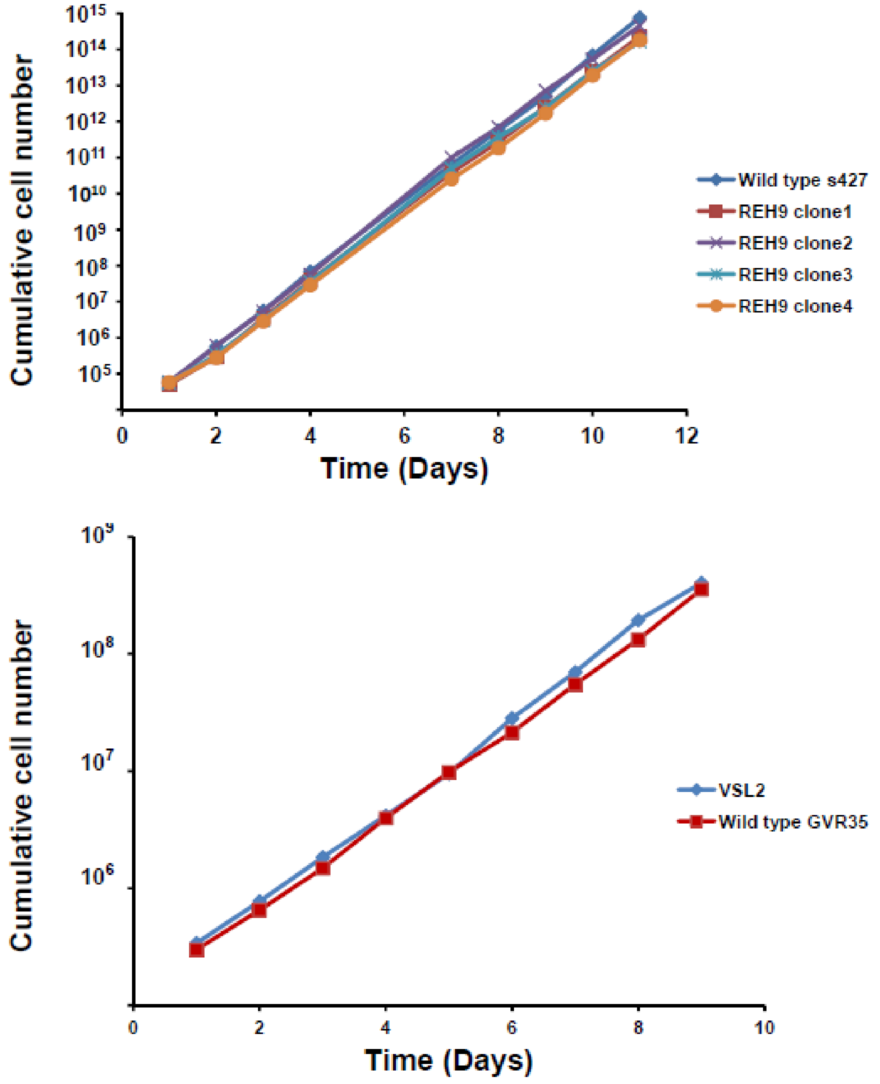

Figure S1. Cumulative growth of cultured bloodstream form *T. brucei* expressing the “humanized” thermostable red-shifted luciferase (Ppy RE9H). Upper graph; *T. brucei* s427 and four independent bioluminescent clones (see Figure 2). Lower graph; *T. brucei* GVR35 and the highly expressing bioluminescent line VSL2 used in most of the imaging experiments. Parasite growth was monitored as outlined in the Materials and Methods.
