# Supplementary material for: Highly Sensitive In Vivo Imaging of Trypanosoma brucei Expressing “Red-Shifted” Luciferase
Source: PLoS Negl Trop Dis. 2013 Nov 21;7(11):e2571. doi: 10.1371/journal.pntd.0002571 (PMC3836995; doi:10.1371/journal.pntd.0002571)
Supplement: Figure S2 — Stability of luciferase expression in the absence of selective pressure. (A) The Ppy RE9H luciferase signal of a T. brucei s427 clone parasite maintained in the presence or absence of 1 µg ml−1 of puromycin. (B) The Ppy RE9H luciferase activity expressed by a T. brucei GVR35 clone. No significant decrease in bioluminescence was observed during this time, showing stable inheritance of luciferase expression. Each experiment was performed in triplicate and values shown are the means ± SD. (PDF) [file pntd.0002571.s002.pdf]

(A)

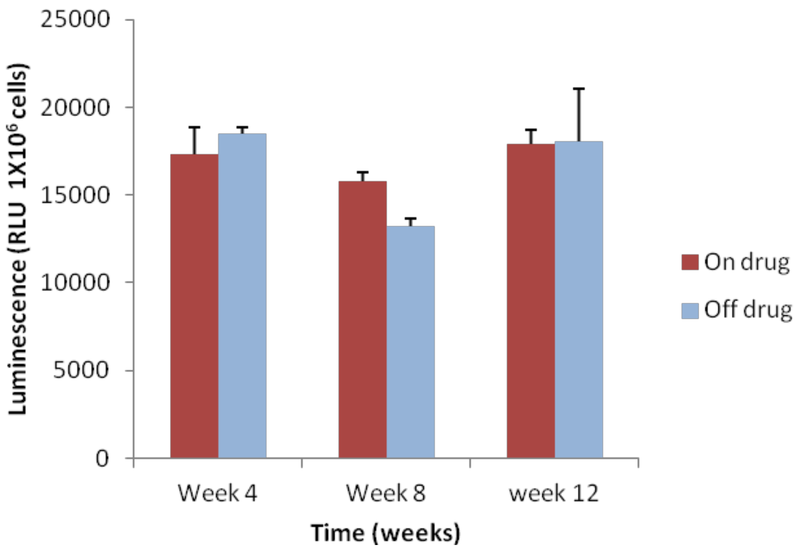

(B)

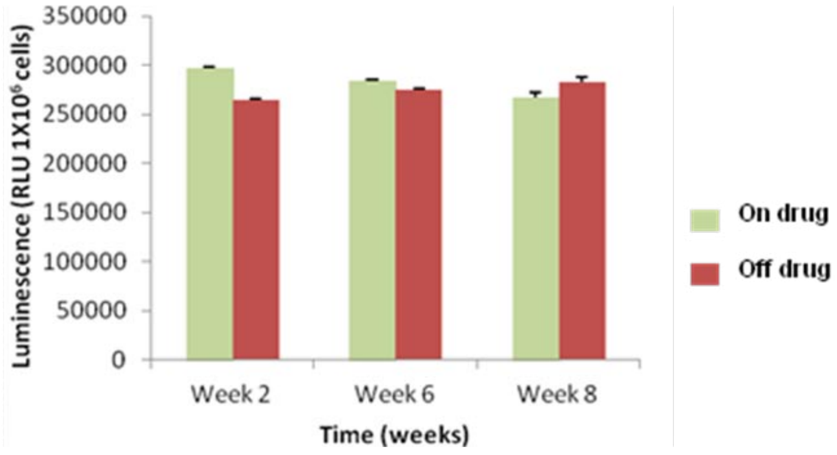

Figure S2. Stability of luciferase expression in the absence of selective pressure. **(A)** The Ppy RE9H luciferase signal of a *T. brucei* s427 clone parasite maintained in the presence or absence of 1 μg ml^{-1} of puromycin. **(B)** The *Ppy RE9H* luciferase activity expressed by a *T. brucei* GVR35 clone. No significant decrease in bioluminescence was observed during this time, showing stable inheritance of luciferase expression. Each experiment was performed in triplicate and values shown are the means ± SD.
