# Supplementary material for: Highly Sensitive In Vivo Imaging of Trypanosoma brucei Expressing “Red-Shifted” Luciferase
Source: PLoS Negl Trop Dis. 2013 Nov 21;7(11):e2571. doi: 10.1371/journal.pntd.0002571 (PMC3836995; doi:10.1371/journal.pntd.0002571)
Supplement: Table S1 — Primer sequences. (PDF) [file pntd.0002571.s003.pdf]

Table S1: Primer sequences

Primer sets	Direction	Enzyme	Sequence
rDNA promoter	Fwd	Sac I	5’-GAGCTCCCTGCAGGCTTTCCACCCAGCGCGGG
	Rev	Not I	5’-GGATCCACAGCTTCGGATTCATATGTG
rDNA target	Fwd	Apa I	5’-GGGCCCGGTAGTTTCCTGCCCTTCTTAT
	Rev	Kpn I	5’-GGTACCTGTTTCGACTAGGGTTCGCTGA
PARP 5’ flank	Fwd	Not I	5’-GCGGCCGCCTGCACGCGCCTTCGAGTTTTTTTTC
	Rev	Xho I	5’CTCGAGGTGAATTTTACTTTTTGGTGTAATTGAAG
VSG 5’ flank	Fwd	Not I	5’-GCGGCCGCACAAGCATTCTATACGTAAAAGATCT
	Rev	Xho I	5’-CTCGAGGCCGCGTTCGTGTCGCGTAGGAATA
Tubulin 3’ flank	Fwd	Bam HI	5’-GGATCCAAAGTGTGACAACGTCGCACCATGTGTA
	Rev	Hind III	5’-AAGCTTAATTCGTTTGGACTATTTTCTTTGATGAAG
Actin 3’ flank	Fwd	Bam HI	5’-GGATCCTGCTTTTAACACCGGGTTGTG
	Rev	Hind III	5’-AAGCTTTGTGCAATACTGCATAGATAA
VSG 3’ flank	Fwd	Bam HI	5’-GGATCCACCCCTCTTTGGCTTGCAGTTTTGC
	Rev	Hind III	5’-AAGCTTCCCCCATTCCTACCTACCTACAAAA
Ppy wild type	Fwd	Xho I	5’-CTCGAGATGGAGGACGCCAAGAACATCA
	Rev	Bam HI	5’-GGATCCTCAGATCTTGCCGCCCTTCTTGGC
Ppy RE9	Fwd	Xho I	5’-CTCGAGATGGAAGACGCCAAAAACATAA
	Rev	Bam HI	5’-GGATCCTTACAATTTGGACTTTCCGCCCTT
Ppy RETS	Fwd	Xho I	5’-CTCGAGATGGAAGACGCCAAAAACATAA
	Rev	Bam HI	5’-GGATCCTTACAATTTGGACTTTCCGCCCTT
Ppy RE9H	Fwd	Xho I	5’-CTCGAGATGGAGGACGCCAAGAACATCA
	Rev	Bam HI	5’-GGATCCTCAGATCTTGCCGCCCTTCTTGGC
